# Guinea pig herpes like virus is a gamma herpesvirus

**DOI:** 10.1007/s11262-024-02054-x

**Published:** 2024-02-10

**Authors:** Brent A. Stanfield, Emmanuelle Ruiz, Vladimir N. Chouljenko, Konstantin G. Kousoulas

**Affiliations:** Division of Biotechnology and Molecular Medicine, Department of Pathobiological Sciences, School of Veterinary Medicine, Louisiana State University, Baton Rouge, LA 70803 USA

**Keywords:** Guinea pig herpes-like virus, GPHLV, Gammaherpesvirus, *Rhadinovirus*, Guinea pig, Genome

## Abstract

Guinea Pig Herpes-Like Virus (GPHLV) is a virus isolated from leukemic guinea pigs with herpes virus-like morphology described by Hsiung and Kaplow in 1969. GPHLV transformed embryonic cells from Syrian hamsters or rats, which were tumorigenic in adult animals. Herein, we present the genomic sequence of GPHLV strain LK40 as a reference for future molecular analysis. GPHLV has a broad host tropism and replicates efficiently in Guinea pig, Cat, and Green African Monkey-derived cell lines. GPHLV has a GC content of 35.45%. The genome is predicted to encode at least 75 open-reading frames (ORFs) with 84% (63 ORFs) sharing homology to human Kaposi Sarcoma Associated Herpes Virus (KSHV). Importantly, GPHLV encodes homologues of the KSHV oncogenes, vBCL2 (ORF16), vPK (ORF36), viral cyclin (v-cyclin, ORF72), the latency associated nuclear antigen (LANA, ORF73), and vGPCR (ORF74). GPHLV is a *Rhadinovirus* of Cavia porcellus, and we propose the formal name of *Caviid gamma herpesvirus 1* (CaGHV-1). GPHLV can be a novel small animal model of *Rhadinovirus* pathogenesis with broad host tropism.

## Introduction

Gammaherpesviruses are a group of medically significant pathogens with global distribution and high prevalence. The two human gammaherpesviruses, Epstein-Barr virus (EBV, Subfamily *Lymphocryptovirus*) and Kaposi’s sarcoma-associated herpesvirus (KSHV, subfamily *Rhadinovirus*) are both capable of malignant transformation of lymphocytes, predominantly B cells, as well as epithelial cells (EBV) and endothelial cells (KSHV), resulting in an array of life-threatening cancers [1–3].

KSHV-encoded oncogenes responsible for induction of cancer are diverse and target several cellular processes [2, 4]. Transformation of lymphocytes and endothelial cells by KSHV occurs due to expression of several viral oncogenes, particularly the latency-associated nuclear antigen (LANA1,ORF73), viral cyclin (v-cyclin, ORF72), and viral FLICE-inhibitory protein (vFLIP, ORF71), the viral G-Protein Coupled Receptor (vGPCR, ORF74), the viral Bcl-2 homologue (ORF16), the viral IL-6 (vIL-6, K2), the viral Interferon Regulatory Factors (vIRF-1/K9, and vIRF-3/K10.6), ORF K1 (K1), ORF K15 (K15), and the viral protein kinase (vPK, ORF36), all of which prevent apoptosis and senescence, manipulate cell cycle regulatory elements, activate survival processes, and induce proliferation [2, 4–10]. Transformed cells are typically kept in check by immune pressures, primarily CD8^+^ T cells, which are able to prevent malignant spread; however, during immunosuppression, both lymphoproliferative and endothelial malignancies may form in KSHV-infected patients [11, 12]. Importantly, KSHV-associated malignancies often consist of both lytically- and latently-infected cells, reducing the efficacy of antiviral drugs which target only lytic viral replication [13].

Herpesviruses are highly species-specific and experimental study of human gammaherpesviruses is largely limited to *in-vitro* work. Animal research on KSHV mostly utilizes murine gammaherpesvirus 4 strain 68 (MHV68), a fellow member of the *Rhadinovirus* genus. This model is limited by the relatively low sequence homology and the physiological differences between mice and humans, but it has nevertheless been the most important animal model of gammaherpesvirus infection for over three decades [14–16]. Improvements of small animal models of human gammaherpesvirus infection is therefore a priority in medical research. Non-human primate models have proven to be the best models of KSHV pathogenesis. These models utilize viruses that are closely related to KSHV and cause similar histopathological changes to that observed with KSHV infection in humans. The Rhesus Rhadinovirus (RRV) model, characterized by the gammaherpesvirus RRV (Macacine gammaherpesvirus 5), offers insights into Kaposi’s Sarcoma (KS) pathogenesis [17]. With genomic similarities to KSHV, RRV has been shown to induce KS-like lesions in rhesus macaques upon co-infection with simian immunodeficiency virus (SIV). These KS-like lesions displayed spindle-shaped cell proliferation however tumors were associated with the peritoneal cavity in infected animals [18]. In contrast, the Retroperitoneal Fibromatosis-Associated Herpesvirus (RFHV) model involves the gammaherpesvirus RFHV (Macacine gammaherpesvirus 8) [19]. This virus was identified in retroperitoneal fibromatosis observed in captive macaques [20]. The genome of RFHV has been determined to be most similar virus to KSHV sequenced to date and encodes homologues to every KSHV gene except ORF11, K5, and K6 [21]. Despite challenges in RFHV isolation and *in-vitro* culture of the virus, experimental infection in macaques leads to KS-like fibrosarcoma in the colon-rectum region with spindle-shaped cell proliferation [22]. Both RRV and RFHV, belonging to the subfamily of gammaherpesviruses *Rhadinoviruses* and share significant genomic and proteomic similarity with KSHV [23]. These viruses demonstrate KS-like lesions in experimentally infected animals which highlights their importance as relevant animal models for studying KSHV-associated malignancies. The observation that lentivirus infection is associated with KS-like lesions in both models further underscores their relevance in understanding the complex pathogenesis of Kaposi’s Sarcoma. However, the overwhelming cost of NHP studies impedes the development of therapeutics and prophylactics targeting KSHV. Pre-clinical development of a low-cost small animal model is needed.

Guinea Pig Herpes Like Virus (GPHLV) was initially isolated from the buffy coat of leukemia susceptible strain 2 guinea pigs and the virus replicated efficiently in rabbit cells [24, 25]. Also, the virus was described as antigenically distinct from guinea pig cytomegalovirus (GPCMV) [24]. GPHLV was first demonstrated to transform primary embryonic Syrian Hamster cells in 1973 [26] and these cells were later shown to be tumorigenic when engrafted into inbred and outbred adult hamsters by intraperitoneal or subcutaneous injection [27]. *J. S. Rhim* later confirmed the transforming capacity of GPHLV in embryonic rat cells which were later shown to be tumorigenic when engrafted in newborn rats. Rhim also extended the *in-vitro* host tropism of GPHLV to include Green African Monkey (Vero) cells, mink, cat, and rat cells [28]. To date, only a comparative restriction fragment length polymorphism analysis has been conducted to describe the genetic difference between GPHLV, GPCMV, and an additional endogenous herpes virus isolated from *Cavia porcellus* (Guinea Pig X Virus, *Caviid Herpesvirus 3*) [29]. Studies of GPHLV ceased in the 1980s, however, as MHV68 became the primary small-animal model for human gammaherpesviruses. Indeed, GPHLV was never formally classified as a gammaherpesvirus. To this end, we sought to better understand GPHLV and to determine its viability in gammaherpesvirus research.

In this paper, we present the first full-genome sequence of GPHLV, demonstrating its phylogenetic relationship to other important gammaherpesviruses in the *Rhadinovirus* subfamily. We further demonstrate that GPHLV shows substantial sequence homology to KSHV in critical loci.

## Materials and methods

### Cell lines and viruses

Green African monkey kidney (Vero) cells, Crandell-Rees Feline Kidney Cell (CRFK), guinea pig 104c1 GPC-16, and JH4 clone 1 were purchased from ATCC (ATCC, Catalogue #s: CCL-81, CCL-94, CRL-1405, CCL-242, and CCL-158, respectively) and maintained in either DMEM (Gibco, Catalogue #: 11965), RPMI-1640 (Gibco, Catalogue #: 11875), or F-12 K (Gibco, Catalogue #: 21127022) containing 10% fetal bovine serum (Hyclone, Catalogue #: SH30071.03HI) and 1 × Penicillin–Streptomycin (Hyclone, Catalogue #: SV30010) at 37 °C and 5% CO_2_ according to ATCC recommendations. Guinea Pig Herpes Like Virus Strain LK40 was purchased from ATCC and referred to in this manuscript as GPHLV (ATCC, Catalogue #: VR-543) and grown to high titer on Vero cells.

### Pan-herpesvirus nested PCR and phylogenetic classification

Total genomic DNA was isolated from Vero cells infected with GPHLV using the Qiagen DNeasy Blood and Tissue kit (Qiagen, Catalogue #: 69504) and subjected to a pan-herpesvirus nested PCR as described by Ehlers et al*.* [30]. Briefly, pooled primers for first round PCR (1 µM final concentration) were added to a reaction containing 10 ng of DNA. Phusion Hot-Start Flex 2 × Master Mix (New England Biolabs, Catalogue #: M0536L) was used and.

### Viral purification and DNA isolation

GPHLV Strain LK40 was grown to high titer on Vero cells. After 8 days of infection and visualization of 100% cytopathic effect, culture flasks were frozen at − 80 °C. Flasks were then thawed at room temperature and culture media collected in 50 mL tubes. Cell debris was pelleted by centrifugation at 3000×*g* for 10 min at 4 °C and the pellet was freeze thawed two additional times before resuspension in the supernatant. Virus was then purified by ultracentrifugation as previously described [31]. Briefly, cellular debris was removed from the supernatant by centrifugation at 9000×*g* for 15 min at 4 °C and the clarified supernatant was passed through a 0.45 µm syringe filter. Filtered supernatant was then floated on a 15% sucrose cushion and centrifuged at 80,000×*g* for 60 min at 4 °C. Viral pellet was resuspended in a minimal volume of 1 × PBS overnight at 4 °C. Resuspended virus was then treated with DNase I (NEB, Catalogue #: M0303S) for 15 min at 37 °C to remove exogenous host cell DNA and inactivated with the addition of 0.5 M EDTA to a final concentration of 5 mM. Additionally, DNase I was heat inactivated at 75 °C for 10 min. Following DNase treatment/inactivation, Virus was lysed with the addition of 10% SDS to a final concentration of 1% and the addition of 2.5 µL of RNase A (NEB, Catalogue #: T3018L) and Proteinase K (NEB, Catalogue #: P8107S) at 56 °C for 5 min. Viral DNA was then isolated by phenol–chloroform extraction and ethanol precipitation.

### *Next generation sequencing, viral classification, *De Novo* assembly, and ORF identification*

Paired-end whole genome DNA sequencing of GPHLV Strain LK40 was performed using Illumina Miseq instrument and 600 cycles MiSeq Reagent Kit v3. DNA concentration for libraries construction was determined using Qubit dsDNA HS Assay Kit. A total of 1 ng of DNA for each sample was used to prepare sequencing libraries using Nextera XT DNA Library Prep Kit and Nextera XT Index Kit v2 to differentiate each sample with individual barcode. Final libraries quality, size and concentration were determined using Fragment Analyzer Instrument. Libraries were mixed with the same molar ratio and run again on Fragment Analyzer to validate pooled samples for the actual sequencing. Raw paired end FASTQ reads were submitted to the Bacterial and Viral Bioinformatics Resource Center (BV-BRC) Taxonomic Classification Tool: https://www.bv-brc.org/app/TaxonomicClassification.

De Novo assembly of Illumina FastQ files was conducted as follows. FastQ files were processed with Trimmomatic [32] for quality control and filtering, with 36, 3, and 3 bases as minimal length, leading and trailing filtering, respectively. De novo transcriptome assembly was performed with Spades [33] and the longest contig (103374 nucleotides) was selected for further analysis. The Tauber bioinformatic platform (T-bio Info) was used for the bioinformatic analyses described previously [34].

The longest contig from the De Novo assembly was annotated using the Prodigal gene prediction software [35] available on github at (https://github.com/hyattpd/Prodigal). Amino acid sequences of predicted ORFs were generated using the Prodigal in FASTA format and blasted against the Herpesviridae (taxid: 10292) using the Blastp tool available from the National Center for Biotechnology Information (https://blast.ncbi.nlm.nih.gov/Blast.cgi?PAGE=Proteins). The viral genome was annotated using the KSHV identification system and virus specific genes annotated with G1-G12. The GPHLV genome has been deposited in GenBank under the Accession Number OQ679822.

## Results

### In-Vitro culture of GPHLV

Previous studies on the *in-vitro* culture of GPHLV have demonstrated the ability of the virus to replicate on Green African Monkey (Vero) cells, mink, cat, guinea pig, and rat cells [28]. Cultures of Vero, CRFK (Feline Kidney), 104c1 (Fetal Guinea pig), JH4 (Guinea pig lung), and GPC-16 (Guinea pig Large intestine/colon) cells were seeded at 5E5 cells/well and infected with 10 uL of ATCC stock GPHLV LK-40. Cells were observed for 7 days and Vero cells were selected to expand the GPHLV stock for their ease of use and the high cytopathic effect observed in this study period (Table 1). High titer cultures and purified viral DNA were prepared as described in the Materials and methods.

**Table 1:** Cell lines used to culture GPHLV

Organism	Cell Line	Tissue	CPE
*Chlorocebus aethiops*	Vero	Kidney	+ + +
*Felis catus*	CRFK	Kidney	+ + +
*Cavia Procellus*	104C1	Fetus	+ +
*Cavia Procellus*	GPC-16	Large intestine/colon	+
*Cavia Procellus*	JH4 Clone 1	Lung	+
*Mus Mucsulus*	NIH-3T3	Embryo	−

Cell lines were selected based on previously published culture techniques in either cell lines or primary tissues derived from the listed organisms

Cytopathic effect (CPR) was scored after 5 days post infection. +  +  + (High CPE), +  + (Moderate CPE), + (Low CPE),),− (No CPE)

### Pan-herpesvirus nested PCR and phylogenetic classification

**Fig. 1:**
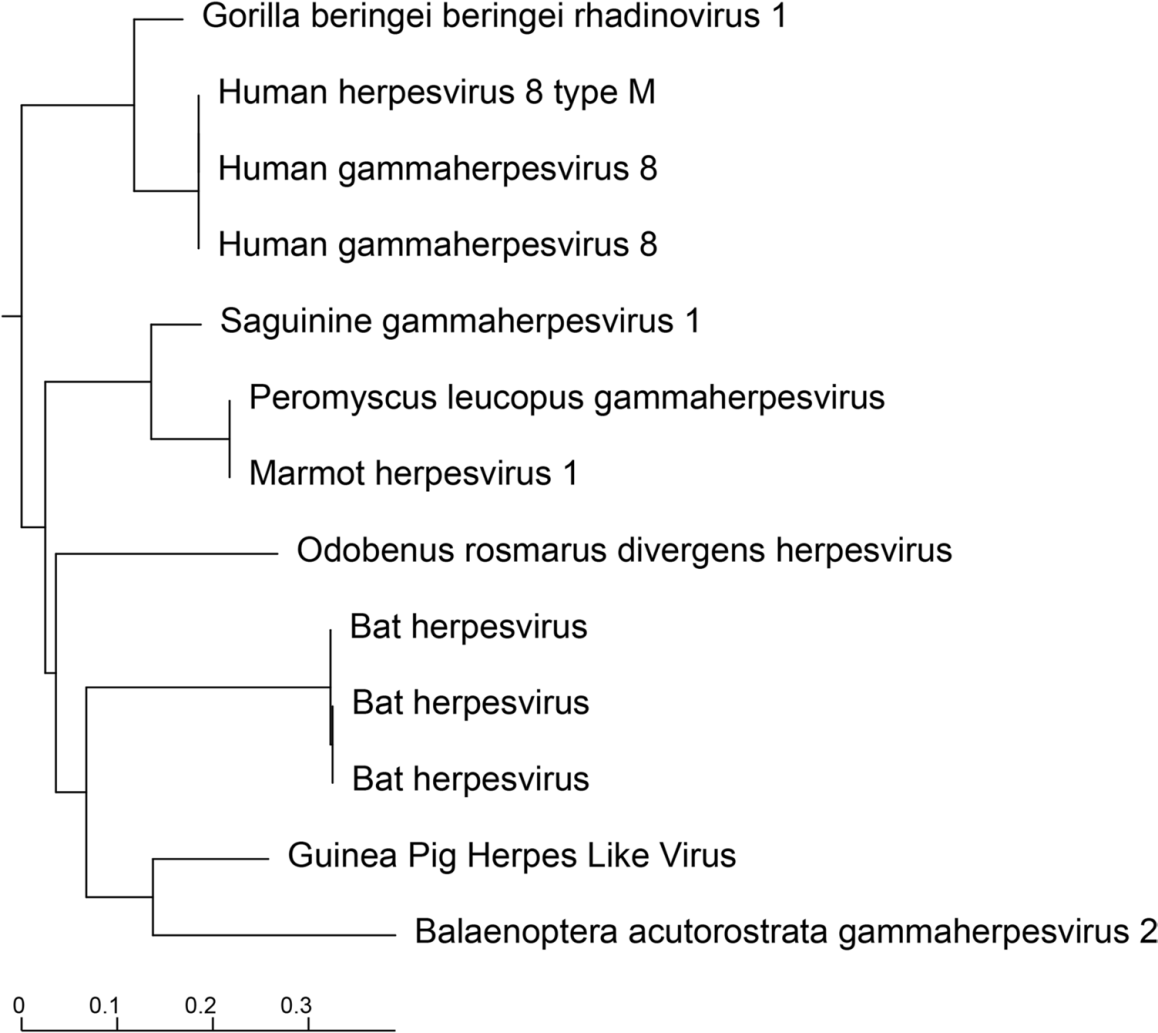
Phylogenetic Analysis of the Translated Nested Pan Herpesvirus PCR Product. The translated Nested PCR product clusters phylogenetically with other viruses in the *gammaherpesvirinae* Subfamily of herpesviruses. Rendered from NCBI BLASTp results using the Newick Display tool

**Fig. 2:**
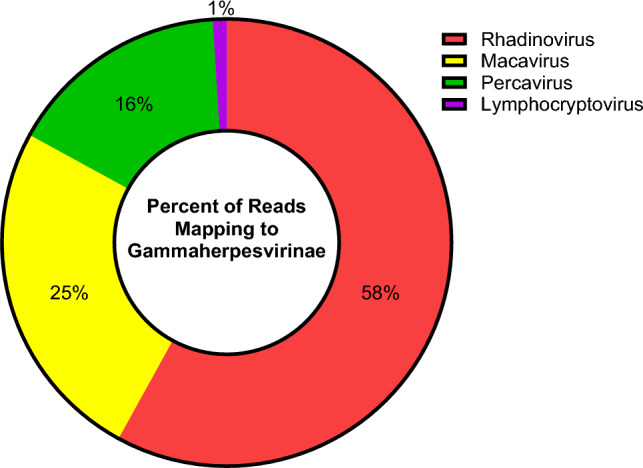
Taxonomic Classification of GPHLV. Raw paired end FASTQ reads were submitted to the BV-BRC Taxonomic Classification Tool. Of the parent classifications 99% of the reads mapped to the *gammaherpesvirinae* subfamily of herpesviruses with 58% of reads identified as belonging to *Rhadinoviruses*

Nested PCR of *Herpesveridae* DNA polymerase was performed as described in Ehlers et al*.* [30]. Second round PCR produced a 219 bp band. This amplicon was then excised from a 1% agarose gel and DNA was isolated for sanger sequencing. A consensus sequence was then constructed from forward and reverse reads which was then translated to its amino acid (AA) sequence. The resulting AA sequence was then submitted to Blastp restricting the search to *Herpesveridae*. The phylogenetic tree generated by the search results is presented in Fig. 1. This small (73 AA) sequence clusters in a group of herpesviruses known to belong to the subfamily *Rhadinovirus* and is most similar to a gammaherpesvirus first identified in the common mink whale (Balaenoptera acutorostrata gammaherpesvirus 2). Further characterization of the GPHLV genome was conducted by Illumina Next Generation Sequencing analysis. Raw FastQ reads were submitted to the BV-BRC Metagenomics Taxonomic Classification tool. 58% of the reads mapping to *Gammaherpesvirinae* identify as belonging to the *Rhadinovirus* subfamily of gammaherpesviruses (Fig. 2).

#### Structure of the GPHLV genome

**Fig. 3:**
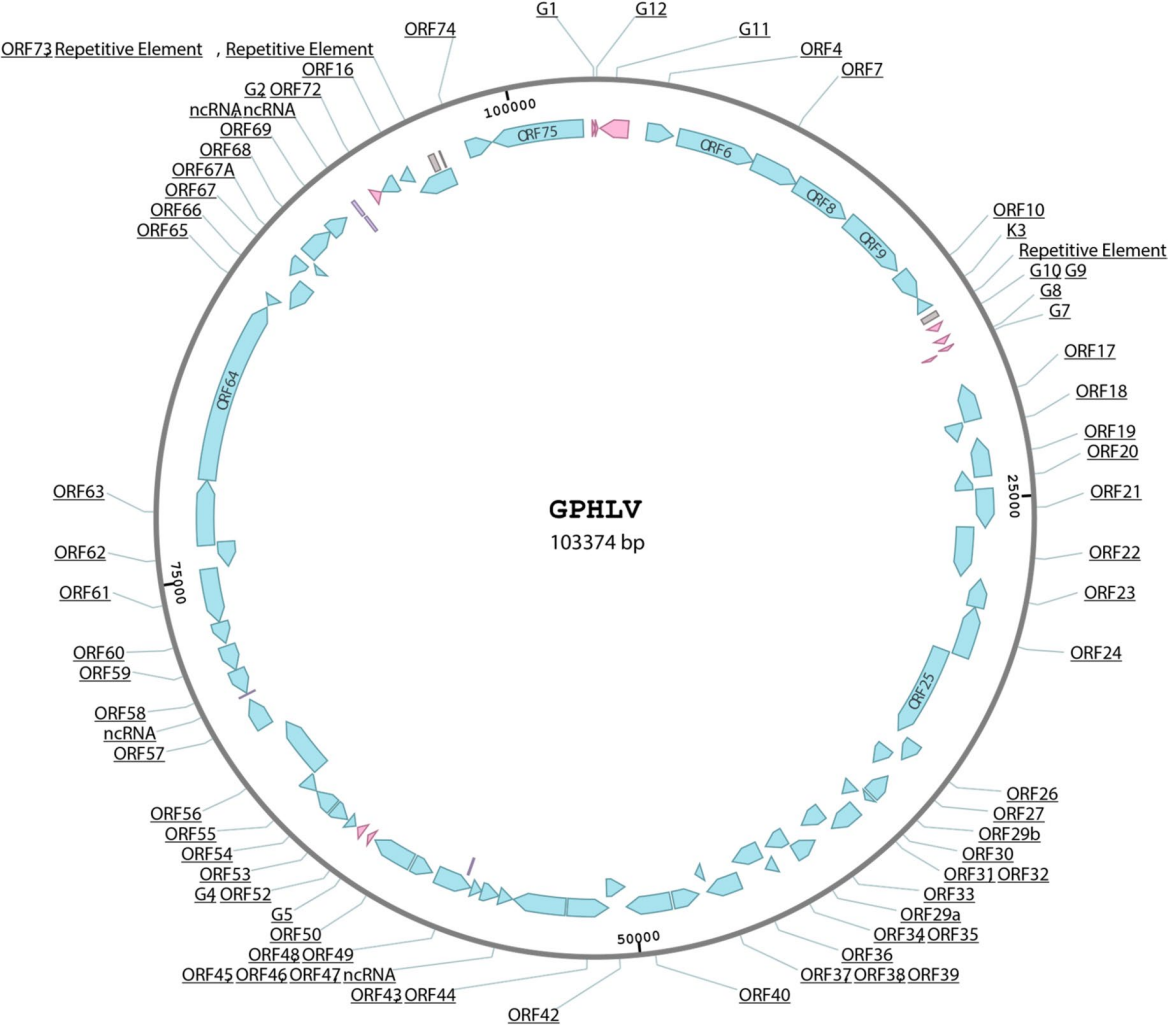
Schematic Representation of the GPHLV genome. Schematic Representation of the GPHLV genome. A 103374 base pair consensus genome was assembled de novo from Illumina NGS reads of DNA isolated from purified GPHLV virions. The genomic structure is homologous to the KSHV genome and places GPHLV in the *Rhadinovirus* subfamily of gammaherpesviruses. 63 of these ORFs share homology to KSHV genes (Blue) with potentially 12 GPHLV specific genes (G1-G12, pink ORFS). The GPHLV genome also encodes several potential ncRNAs (Purple) and repetitive elements (Gray). GenBank: OQ679822.1. GPHLV has a GC content of 35.45%

**Fig. 4:**
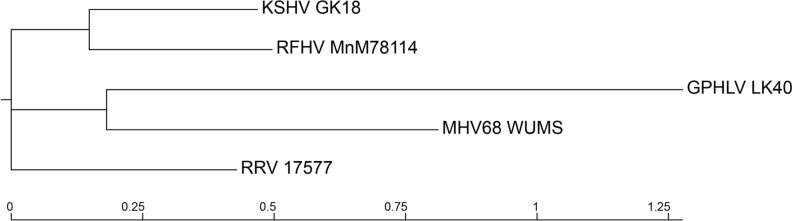
Phylogenetic Tree of *Rhadinovirus* Model Organisms. Phylogenetic analysis of the complete nucleotide sequences of select *Rhadinovirus* genomes was conducted using the BV-BRC Viral Genome Tree tool. Reference genomes for KSHV (GK18), RFHV (MnM78114), MHV68 (WUMS), and RRV (17577) were utilized to analyze the phylogenetic distribution of the GPHLV genome in comparison to viruses used to model *Rhadinovirus* pathogenesis

Genomic viral DNA was purified as previously described [31] from high titer virus grown on green African monkey kidney cells (Vero cells). DNA was then utilized for next generation sequencing library preparation and analysis as described in the materials and methods. A consensus genome was assembled via de novo assembly yielding a 103374 base pair sequence. An unbiased open reading frame prediction was applied to this consensus identifying 75 major open reading frames 63 of which bare sequence homology to human Kaposi’s Sarcoma Associated Herpes Virus (KSHV) and has a GC content of 35.45% (Figs. 3, 4).

#### Analysis of GPHLV predicted major ORFs

**Fig. 5:**
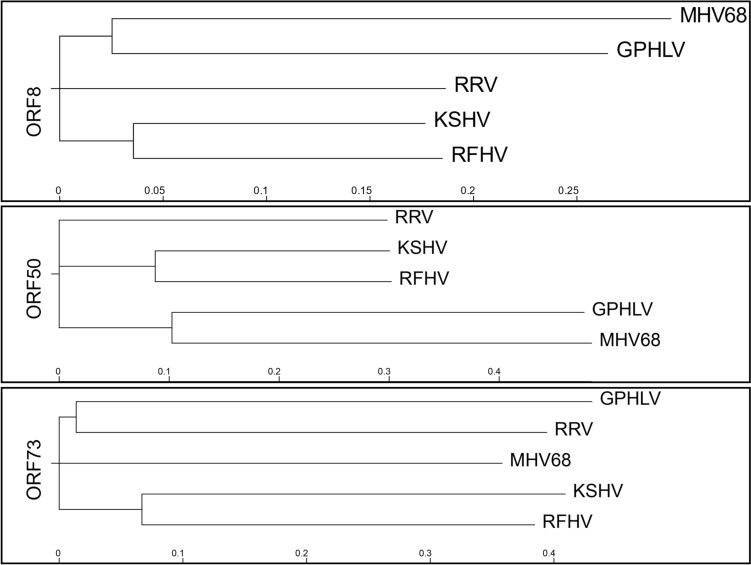
Phylogenetic Analysis of Key *Rhadinovirus* ORFs. Phylogenetic trees for translations of individual ORFs were constructed using Clustal Omega. ORF8 (Glycoprotein B), ORF50 (RTA), and ORF73 (LANA) were selected due to their conserved importance in the *Rhadinovirus* lifecycle. Scale bars represent phylogenetic distance

**Fig. 6:**
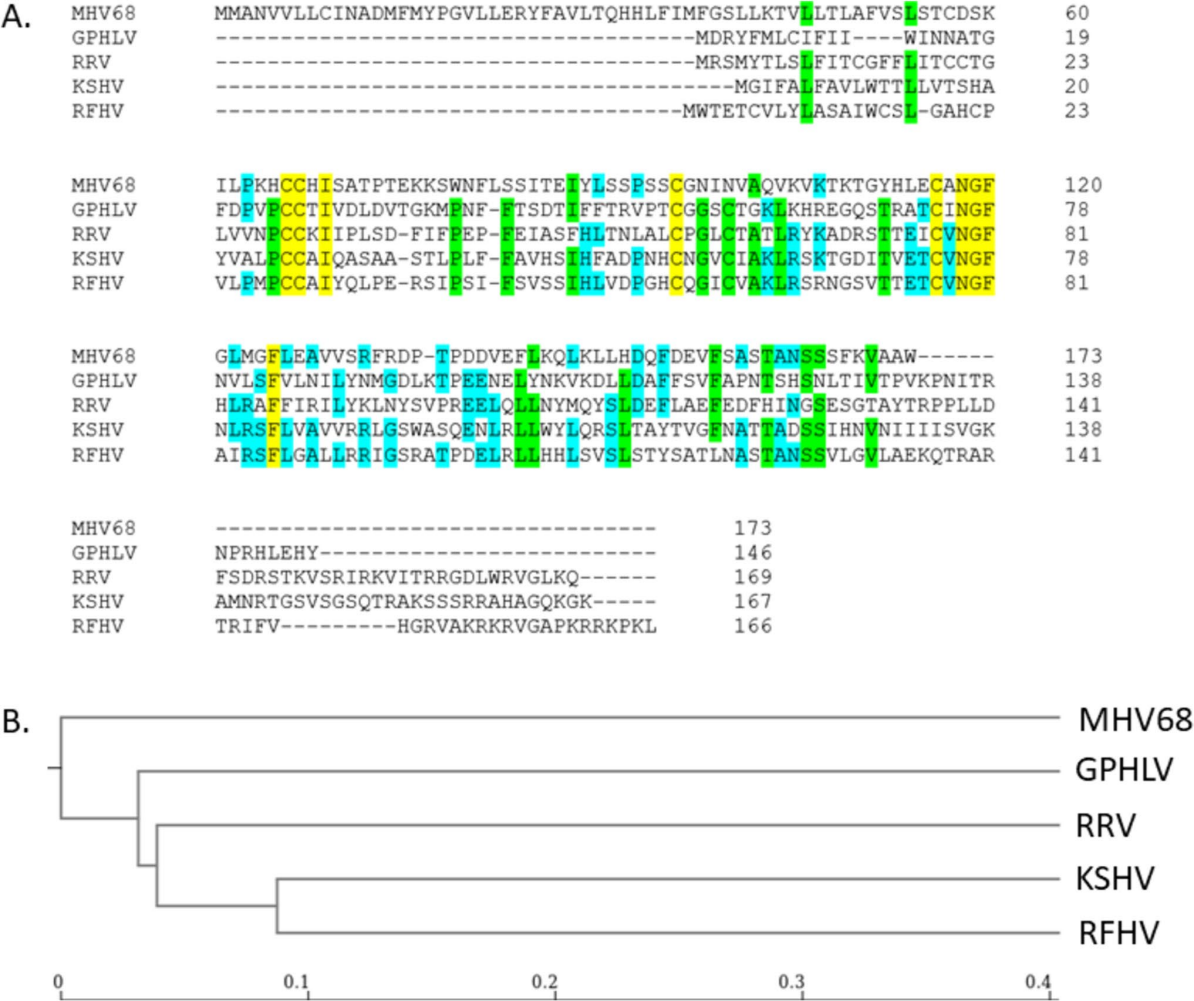
Sequence Conservation of Glycoprotein L. A. Clustal Omega alignment of MHV68, GPHLV, KSHV, RRV, and RFHV glycoprotein L (gL) amino acid sequences. Residues highlighted in yellow are 100% conserved, green 80%, and blue 60% between the 5 viruses. B. Phylogenetic analysis of gL generated by Clustal Omega and visualized by the Newick Display tool

Putative ORFs were identified using an open-source dynamic programming genefinding algorithm (Podigal: https://github.com/hyattpd/Prodigal). Predicted ORFs were exported in FASTA format and blasted against the *Herpesviridae* nonredundant protein database on NCBI. Significant homology was identified between 63 of the 75 identified ORFs. A comparison of the GPHLV proteome utilizing the Proteome Comparison Tool which is available through the Bacterial and Viral Bioinformatics Resource Center (BV-BRC: https://www.bv-brc.org/) was used to compare the proteomes of viruses used in current animal models of KSHV pathogenesis. Retroperitoneal fibromatosis-associated herpesvirus (RFHV), Macacine gammaherpesvirus 5 (RRV), Murid gammaherpesvirus 4 (MHV68), and Guinea Pig Herpes Like Virus (GPHLV) were compared to human KSHV (Table 2). Proteins with identities less than 10% homology or with coverages less than 30% were excluded from this analysis. This analysis demonstrates GPHLV as having significantly more proteomic homology to KSHV than MHV68. Of particular interest is the high level of conservation observed between GPHLV lytic antigens glycoprotein B (gB, ORF8), glycoprotein H (gH, ORF22), and glycoprotein L (gL, ORF47) with their orthologous KSHV genes (56.5%, 32.7%, and 29.6% respectively). GPHLV gL shares similar conservation as RRV to KSHV gL (29.6% vs 29.3% respectively). This could suggest a potential for GPHLV to maintain conserved entry mechanisms and tissue tropisms with KSHV as observed with RRV. More studies are needed to assess GPHLV tropism in-vivo. Additionally, the predicted GPHLV replication transactivator (RTA, ORF50), gB, and ORF73 demonstrate interesting conservation with well characterized preclinical *Rhadinovirus* models (Fig. 5). Clustal Omega analysis of gL identifies conserved amino acid residues and phylogenetically clusters GPHLV more closely to KSHV, RRV, and RFHV than MHV68 (Fig. 6).

**Table 2:** Proteomic comparison of RFHV, RRV, MHV68, and GPHLV to KSHV

KSHV Gene	RFHV percent identity	RRV percent identity	MHV68 percent identity	GPHLV percent identity	KSHV gene	RFHV percent identity	RRV percent identity	MHV68 percent identity	GPHLV percent identity
K1^a^	28.10%				ORF 40	43.90%	33.20%	26.00%	24.20%
ORF 4	39.90%	44.10%	28.50%	33.20%	ORF 42	60.20%	46.80%	36.80%	37.50%
ORF6	71.50%	63.20%	40.60%	47.50%	ORF 43	75.70%	63.70%	48.20%	56.00%
ORF7	58.90%	52.70%	34.30%	39.70%	ORF 44	74.40%	65.80%	54.70%	57.10%
ORF8	71.30%	66.70%	53.70%	56.50%	ORF 45	40.20%	37.30%		
ORF9	75.70%	67.10%	56.60%	55.30%	ORF 46	69.00%	59.80%	53.60%	53.80%
ORF 10	44.60%	34.00%	23.00%	18.90%	ORF 47	47.70%	29.30%	28.90%	29.60%
ORF 11		33.80%			ORF 48	36.20%	32.50%	23.40%	23.90%
K2^a^	34.50%				ORF 49	64.20%	54.30%	21.90%	22.40%
ORF2	57.90%	46.50%			ORF 50	55.20%	55.70%		28.70%
K3	41.50%				K8	31.80%			
ORF 70	74.00%	73.80%			K8.1				
K4	60.60%	44.10%			ORF 52	55.00%	46.20%	31.50%	38.30%
K4.1	55.40%				ORF 53	71.70%	61.50%	37.50%	34.50%
K4.2					ORF 54	50.00%	41.80%	32.50%	33.80%
K5	39.80%		29.70%		ORF 55	75.90%	60.20%	42.40%	40.50%
K6	51.10%	37.50%			ORF 56	58.40%	52.40%	36.20%	37.30%
K7					ORF 57	55.20%	44.70%		30.80%
ORF 16^a^	42.30%	49.70%			K9^a^	40.10%	28.40%		
ORF 17	48.40%	42.80%	35.00%	36.00%	K10				
ORF 17.5	34.50%	32.20%	29.80%	27.70%	K10.6^a^				
ORF 18	66.10%	58.00%	41.50%	43.20%	K11	41.30%			
ORF 19	59.50%	52.80%	36.10%	42.10%	ORF 58	52.60%	38.70%	21.70%	30.40%
ORF 20	51.80%	48.00%	34.30%	42.30%	ORF 59	58.10%	52.20%	36.10%	26.00%
ORF 21	54.60%	44.50%	30.00%	35.90%	ORF 60	79.70%	69.80%	61.30%	63.60%
ORF 22	49.90%	40.80%	29.40%	32.70%	ORF 61	71.40%	65.70%	47.40%	49.90%
ORF 23	53.90%	48.40%	25.40%	34.40%	ORF 62	65.90%	56.50%	28.20%	34.00%
ORF 24	66.30%	59.20%	41.50%	44.20%	ORF 63	44.90%	42.70%	23.90%	26.90%
ORF 25	82.10%	72.40%	56.80%	61.20%	ORF 64	47.60%	38.40%	25.80%	27.70%
ORF 26	78.30%	65.10%	41.90%	45.70%	ORF 65	46.60%	43.80%		37.80%
ORF 27	44.80%	28.90%		27.20%	ORF 66	57.90%	47.30%	31.50%	38.30%
ORF 28	51.00%				ORF 67	64.00%	66.10%	43.10%	50.80%
ORF 29	72.10%	66.40%	49.60%	57.10%	ORF 67A	60.80%	66.70%		43.10%
ORF 30		44.30%	34.50%	43.30%	ORF 68	60.50%	48.00%	33.00%	37.90%
ORF 31	67.70%	48.70%	38.00%	35.40%	ORF 69	68.10%	61.70%	46.20%	42.30%
ORF 32	44.00%	40.60%	24.00%	26.00%	K12				
ORF 33	60.90%	42.90%	34.30%	34.60%	ORF 71^a^	54.90%	33.30%		
ORF 34	60.10%	49.20%	30.40%	39.20%	ORF 72^a^	47.30%	41.00%	31.80%	34.40%
ORF 35	56.00%	37.90%	28.60%	34.70%	ORF 73^a^				
ORF 36^a^	67.10%	46.20%	27.50%	30.10%	K14	40.90%	36.40%		
ORF 37	72.20%	63.70%	44.70%	47.60%	ORF 74^a^	56.70%	44.40%	24.80%	26.90%
ORF 38	52.40%	47.40%		28.60%	ORF 75	62.20%	43.60%	27.70%	28.60%
ORF 39	66.00%	59.50%	51.50%	47.40%	K15^a^	24.40%			

The Proteome Comparison Tool which is available through the BACTERIAL AND VIRAL BIOINFORMATICS RESOURCE CENTER (BV-BRC) was used to compare the proteomes of viruses used in current animal models of KSHV pathogenesis: Retroperitoneal fibromatosis-associated herpesvirus (RFHV), Macacine gammaherpesvirus 5 (RRV), Murid gammaherpesvirus 4 (MHV68), and Guinea Pig Herpes Like Virus (GPHLV) were compared to human KSHV

Percent Identity is calculated relative to the homologous KSHV protein

Proteins with identities less than 0.1 and a coverage of 0.3 were excluded from the analysis

^a^Denotes known KSHV oncogenes

## Discussion

Twenty percent of all human cancers have a viral etiology [36]. These oncoviruses share several traits that are consistently observed. (1) Viral cancers are observed in the context of persistent infections. (2) These cancers occur many years to decades after acute viral disease. (3) The immune system can play an immunopathogenic or immunoprotective role in the development of viral associated cancers [37]. Classic Kaposi’s sarcoma (KS) was first described in 1872 by Moritz Kaposi as an aggressive idiopathic multi-pigmented skin sarcoma identified in elderly European men, all of whom died within 2 years [38]. Kaposi’s sarcoma-associated herpesvirus (KSHV) is a medically important oncovirus which is the causative agent of KS, multicentric Castleman’s disease, and primary effusion lymphoma [39]. Development of an effective vaccine to control KSHV and thus reduce the global cancer burden is of high importance and a public health priority [40]. Currently, preclinical development of promising KSHV vaccines is limited without a homologous small animal model of disease. Here, we sequenced a novel herpes virus, Guinea Pig Herpes Like Virus (GPHLV). GPHLV is taxonomically related to KSHV, and presents similar in-vivo features of KSHV associated disease including malignant transformation of B cells [24, 25] and the ability to induce sarcoma like tumors in multiple in-vivo animal models [27, 28]. We sequenced the GPHLV genome and demonstrated that it belongs to the *Rhadinovirus* genus with high proteomic similarity to KSHV in comparison to current models of *Rhadinovirus* pathogenesis (Table 2). GPHLV is a novel gamma herpesvirus suitable to investigate viral oncogenesis in a small animal model which may serve as an effective model for the preclinical development of vaccines and therapeutics against oncogenic herpesviruses.

Current animal models of KSHV have limitations that make it difficult to fully understand the virus and its associated diseases. For instance, rodent models lack the necessary receptor for KSHV entry and are unable to replicate the full range of clinical disease particularly KS like lesions. Non-human primate models, while susceptible to KSHV, do not recapitulate human diseases and are resource-intensive leading to limited numbers of experimentally infected animals and overwhelming study costs [41]. Existing humanized mouse models also fall short of accurately reproducing KSHV-associated malignancies as the engraftment of human fetal tissues into these animals is highly variable, the virus is only able to infect the engrafted immune system, and infection does not result in cellular transformation, excluding the development of a KS model [42]. The limitations of current models underscore the need for innovative approaches, such as developing new comparative models in low-cost model organisms.

Murine gammaherpesvirus 68 (MHV68) is an endemic virus of rodents and was originally isolated from yellow-necked mice and bank voles [14]. MHV68 infection in laboratory mice is a commonly used animal model for studying gammaherpesvirus pathogenesis, including KSHV. However, it is not an ideal model for KSHV-associated malignancy as mice infected with MHV68 infrequently develop lymphoma and have yet to demonstrate KS like lesions [43]. MHV68 infection in mice can cause lymphoproliferative disease, however, in humans Primary Effusion Lymphoma and Multicentric Castleman’s Disease are very rare and primarily occur as Human Immunodeficiency Virus (HIV) associated cancers [44]. This makes it more difficult to extrapolate results from MHV68 studies to human KSHV infections. Furthermore, though mononucleosis is a hallmark of gammaherpesvirus infection, the resulting T cells expanding in response to MHV68 infection in mice are not reactive to MHV68 epitopes [45]. These differences make it difficult to accurately model the immune response to KSHV in humans and limit the translatability of results obtained from MHV68 studies. Therefore, while MHV68 can provide some insights into KSHV pathogenesis, alone it is not a suitable model for studying KSHV-associated malignancy. Multiple animal models are needed to describe the biology of oncogenic gammaherpesvirus infections [46].

Currently, the gold standard of preclinical models for KSHV-like pathogenesis utilize NHP infection with either RRV or retroperitoneal fibromatosis associated herpesvirus (RFHV) as experimental infection with either of these viruses have demonstrated KSHV-like disease. RRV infection of rhesus macaques has demonstrated multicentric Castleman’s disease like B-cell lymphoproliferative disease [18], B-cell lymphoma [47], KS-like tumors [18], and persistent infection [48]. Furthermore, RFHV is the most similar virus to KSHV identified to date [21] and is considered the etiological agent of retroperitoneal fibromatosis (RF, a KS like lesion originating in the retroperitoneum and not the skin) [20]. Experimental infection of NHPs with RFHV has been difficult as the virus does not replicate in culture, however virus isolated from the saliva of naturally infected macaques was able to establish infection in naïve animals [49]. Given the high cost of these studies in NHPs, development of improved small animal models is needed. Further investigation of GPHLV may yield a useful small animal model for the preclinical development of therapeutics and vaccines against oncogenic gammaherpesviruses.

The sequence homology analysis presented in this study provides crucial insights into the potential of GPHLV as a valuable model for studying oncogenic gammaherpesvirus pathogenesis. By comparing the genomic sequence of GPHLV with that of known gammaherpesviruses, particularly KSHV, we have gained a better understanding of the genetic relatedness and potential functional similarities between these viruses. This analysis serves as a foundation for justifying the further development of GPHLV as a relevant model for studying gammaherpesvirus infections and associated malignancies. Here, we identified a significant proportion of GPHLV predicted open reading frames (ORFs) shared homology with those of KSHV, specifically 84% (63 out of 75 ORFs) showed significant sequence similarity. This level of sequence homology is a key finding, as it suggests a close evolutionary relationship between GPHLV and KSHV. Notably, this similarity is not restricted to a few specific genes, but rather spans across a wide array of ORFs, including important oncogenic factors such as latency-associated nuclear antigen (LANA, ORF73), viral cyclin (v-cyclin, ORF72), and the replication transactivator (RTA, ORF50) among others. The significant degree of proteomic homology, especially in critical loci such as glycoprotein B (gB), glycoprotein H (gH), glycoprotein L (gL), and RTA, provides strong evidence that GPHLV and KSHV may share common molecular mechanisms in terms of entry, cell tropism, and lytic replication. Notably, these proteins play pivotal roles in the interaction of the virus with host cells, cell-to-cell spread, immune evasion, and virus replication, suggesting that GPHLV may utilize similar strategies as KSHV for infection and pathogenesis [50]. Further molecular characterization of GPHLV is warranted to assess viral tropism and its association with cellular transformation.

The close genetic relatedness observed between GPHLV and KSHV is particularly significant in the context of model development for KSHV-associated diseases of which there is a significant unmet need [40]. The challenges of studying KSHV in traditional animal models such as mice have been well-documented, primarily due to the low sequence homology and physiological differences between murine gammaherpesvirus 68 (MHV68) and human KSHV. Furthermore, GPHLV’s ability to transform cells and induce tumorigenicity in animal models, as demonstrated in previous studies [26–28], aligns well with the capacity of KSHV to induce malignancies in-vivo. These shared features between GPHLV and KSHV underscore the potential of GPHLV as a relevant small animal model for exploring the mechanisms of gammaherpesvirus-induced oncogenesis.

In conclusion, the comprehensive sequence homology analysis presented in this study highlights the substantial genetic similarity between GPHLV and KSHV. This genetic relatedness, coupled with GPHLV’s tumorigenic capabilities, establishes a strong rationale for considering GPHLV as a biologically relevant model for investigating oncogenic gammaherpevirus pathogenesis, thereby contributing to the advancement of our understanding of gammaherpesvirus-associated diseases and the development of novel therapeutic strategies. Further studies utilizing the GPHLV model have the potential to uncover critical insights into the complex interplay between the virus and host, shedding light on the mechanisms of oncogenesis and facilitating the translation of this knowledge into clinical applications.

## Data availability

Original FASTQ files for this study are available upon request. The consensus genome for GPHLV Strain LK40 has been deposited in NCBI Nucleotide collection under the GenBank under the Accession Number OQ679822.
